# Correction

**DOI:** 10.1111/pbi.14374

**Published:** 2024-05-28

Correction for article ‘Rice‐produced classical swine fever virus glycoprotein E2 with herringbone‐dimer design to enhance immune responses’

Xu, Q. , Ma, F. , Yang, D. , Li, Q. , Yan, L. , Ou, J. , Zhang, L. *et al*. (2023) Rice‐produced classical swine fever virus glycoprotein E2 with herringbone‐dimer design to enhance immune responses. Plant Biotechnol. J., 21, 2546–2559.37572354 10.1111/pbi.14152PMC10651154

The authors want to rearrange the institutions of the authors for change of position. The correct order of author names and affiliations is as follows.

Qianru Xu^1,2,3,†^, Fanshu Ma^2,4,†^, Daichang Yang^5,6^, Qingmei Li^1^, Liming Yan^7^, Jiquan Ou^6^, Longxian Zhang^2,8^, Yunchao Liu^1^, Quan Zhan^6^, Rui Li^1^, Qiang Wei^1^, Hui Hu^2^, Yanan Wang^1^, Xueyang Li^2^, Shenli Zhang^2^, Jifei Yang^1^, Shujun Chai^1^, Yongkun Du^2^, Li Wang^1^, Erqin Zhang^2,8,*^ and Gaiping Zhang^1,2,8,9,*^

^1^Henan Provincial Key Laboratory of Animal Immunology, Henan Academy of Agricultural Sciences, Zhengzhou, China

^2^International Joint Research Center of National Animal Immunology, College of Veterinary Medicine, Henan Agriculture University, Zhengzhou, China

^3^School of Basic Medical Sciences, Henan University, Kaifeng, China

^4^CAS Key Laboratory of Nano‐Bio Interface, Suzhou Institute of Nano‐Tech and Nano‐Bionics, Chinese Academy of Sciences, Suzhou, China

^5^College of Life Science, Wuhan University, Wuhan, China

^6^Wuhan Healthgen Biotechnology Corp., Wuhan, China

^7^Laboratory of Structural Biology, School of Medicine, Tsinghua University, Beijing, China

^8^Longhu Laboratory, Zhengzhou, China

^9^School of Advanced Agricultural Sciences, Peking University, Beijing, China

The published article has also been corrected to reflect the changes.

We apologize for this error.

